# Immersive Virtual Reality Cognitive Stimulation for Delirium Prevention in Older Surgical Intensive Care Unit Patients With Cognitive Impairment: Protocol for a 3-Arm Randomized Pilot Trial

**DOI:** 10.2196/105624

**Published:** 2026-11-05

**Authors:** Hina Faisal, Kenneth Podell, Austin Shelton, Jiaqiong Xu, Malaz Boustani, George Taffet

**Affiliations:** 1 Department of Surgery, Houston Methodist, 6550 Fannin Street, Suite SM1661, Houston, TX, 77030, United States, 1-346-238-0343; 2 Center for Critical Care, Houston Methodist, Houston, TX, United States; 3 Stanley H. Appel Department of Neurology, Houston Methodist, Houston, TX, United States; 4 The Bookout Center, Houston Methodist Academic Institute, Houston, TX, United States; 5 Center for Health Data Science and Analytics, Houston Methodist Research Institute, Houston, TX, United States; 6 Department of Medicine, Indiana University School of Medicine, Indianapolis, IN, United States; 7 Center for Aging Research, Indiana University School of Medicine, Indianapolis, IN, United States; 8 Regenstrief Institute Inc., Indianapolis, IN, United States; 9 Center for Health Innovation and Implementation Science, Indiana University School of Medicine, Indianapolis, IN, United States; 10 Eskenazi Health, Health and Hospital Corporation, Indianapolis, IN, United States; 11 Department of Medicine, Division of Geriatrics, Houston Methodist Hospital, Indianapolis, IN, United States

**Keywords:** delirium, virtual reality, cognitive stimulation, older adults, postoperative delirium, intensive care unit, feasibility trial, digital health, JMIR Research Protocols

## Abstract

**Background:**

Postoperative delirium is common among older adults after major surgery, especially those with cognitive impairment, and is associated with prolonged hospitalization, functional decline, mortality, and subsequent cognitive deterioration. Nonpharmacologic delirium-prevention strategies are recommended but are difficult to deliver reliably in high-acuity environments because they require repeated staff-intensive interactions, consistent patient engagement, and integration into complex intensive care unit (ICU) workflows.

**Objective:**

This study aims to evaluate the feasibility, usability, safety, acceptance, and adherence of ReCognitionVR 2.0, an immersive virtual reality (VR)–based cognitive stimulation intervention designed for older surgical ICU patients with mild-to-moderate cognitive impairment.

**Methods:**

This is a single-center, 3-arm, parallel-group randomized pilot trial. Ninety adults aged 65 years or older undergoing major surgery with expected postoperative ICU admission and Montreal Cognitive Assessment scores of 15 to 24 will be randomized in a 1:1:1 ratio to ReCognitionVR 2.0 cognitive stimulation, passive VR control exposure, or standard-of-care nurse-led reality orientation. VR sessions will be delivered once daily for up to 20 minutes on postoperative days 1 through 3 when participants are calm and alert. The primary outcomes are feasibility metrics, including recruitment, outcome completion, safety and tolerability, usability, acceptance, and adherence. Secondary outcomes estimate preliminary changes in attention and executive function using the Months Backward Test and NIH Toolbox Flanker Inhibitory Control and Attention Test. Postoperative delirium incidence is an exploratory clinical outcome.

**Results:**

This study was funded in September 2025. Participant recruitment and data collection are currently ongoing. Preliminary data quality assessments and exploratory reviews of the collected data are underway. Formal statistical analyses of the trial outcomes will be conducted after participant enrollment is completed and the study database has been finalized and locked. As of July 29, 2026, we had enrolled 18 participants. Early operational experience informed the refinement of exploratory VR performance metrics and the documentation of protocol deviations. Following an oxygen-connection near miss, we added a preintervention oxygen/readiness checklist as an operational safety safeguard. Enrollment of 90 participants is projected to be completed in 2027 if the current recruitment pace is sustained. Results are expected to be published in winter 2027.

**Conclusions:**

This protocol will generate data on feasibility, safety, usability, adherence, digital performance, cognitive assessment, and delirium measurement, all needed to optimize ReCognitionVR 2.0 and to design a future multicenter efficacy trial of VR-enabled delirium prevention in critical care.

## Introduction

Delirium is an acute disturbance of attention and awareness that occurs frequently after major surgery and during critical illness [1-5]. Older adults with preexisting cognitive impairment are particularly vulnerable to surgical stress, pain, sedatives, sleep disruption, inflammation, sensory deprivation, and environmental disorientation that converge during the early postoperative intensive care unit (ICU) period [1-3]. Delirium is associated with increased mortality, prolonged hospitalization, institutionalization, functional decline, and long-term cognitive deterioration [6-11]. Preventing delirium in older surgical ICU patients is therefore a major priority in geriatric critical care [12,13].

Nonpharmacologic strategies are central to delirium prevention. Multicomponent delirium-prevention programs and ICU liberation bundles incorporate reality orientation, early mobilization, sleep promotion, sensory optimization, cognitive engagement, and family participation [12-14]. However, implementation remains inconsistent. Delivery depends on staffing availability, training, competing clinical priorities, and patients’ ability to engage during recovery from major surgery [15,16]. In postoperative ICU settings, patients may be fatigued, in pain, tethered to devices, and exposed to frequent interruptions; these factors limit reliable delivery of traditional cognitive stimulation.

Cognitive stimulation is a promising nonpharmacologic strategy because it targets attention, working memory, orientation, and engagement, all of which are relevant to delirium vulnerability [17-19]. Conventional approaches include reality orientation, workbooks, structured conversation, and therapist- or nurse-delivered activities [20-24]. These approaches can be valuable but are difficult to individualize, may become repetitive, and are challenging to scale in high-acuity environments. Digital cognitive stimulation offers potential advantages by standardizing intervention content, supporting consistent delivery, and generating objective performance data [25-30].

Immersive virtual reality (VR) provides a distinctive platform for digital cognitive stimulation. By combining visuospatial immersion, auditory input, structured orientation, and interactive tasks, VR may increase engagement while reducing environmental distraction [26]. For postoperative ICU patients, a low-movement bedside VR intervention may provide cognitive stimulation without requiring ambulation, extensive staff-mediated activity, or a complex setup. VR also enables capture of human factors and behavioral metrics, including session duration, task accuracy, reaction time, performance trends, tolerance, and adherence [26,31].

ReCognitionVR was initially developed as an immersive VR platform to deliver cognitive stimulation and orientation to hospitalized older adults. Preliminary studies in healthy older volunteers and low-risk geriatric surgical patients suggested that VR-based cognitive stimulation can be usable, tolerable, and acceptable [32,33]. The next translational step is to evaluate whether this approach can be delivered safely and feasibly in a higher-risk population: older adults with mild-to-moderate cognitive impairment after major surgery and ICU admission.

The objective of this pilot randomized trial is to evaluate a 3-arm study comparing ReCognitionVR 2.0 cognitive stimulation, passive VR exposure, and standard-of-care nurse-led reality orientation. The trial is not powered to establish clinical efficacy. Instead, it is designed to estimate recruitment, retention, data completion, intervention adherence, usability, acceptance, safety, variability in cognitive outcomes, feasibility of delirium ascertainment, and the value of VR-derived digital performance metrics. These data will support intervention refinement and planning for a future definitive multicenter trial.

## Methods

### Study Design

This is a single-center, 3-arm, parallel-group randomized pilot trial conducted in surgical ICUs at Houston Methodist Hospital in Houston, Texas. Participants will be randomized in a 1:1:1 ratio to ReCognitionVR 2.0 cognitive stimulation games, passive VR control exposure, or standard-of-care nurse-led reality orientation. All participants will continue to receive usual postoperative and ICU care.

The protocol is PRO00041199, modified version 2 dated April 13, 2026. The protocol lists US Food and Drug Administration investigational device exemption G260027 and classifies the study as a nonsignificant risk device study under 21 CFR 812.3(m). The trial is a behavioral and device-enabled feasibility study. It does not involve an investigational drug, biologic, dietary supplement, or collection of biological specimens.

The study is designed as a pilot and feasibility trial rather than an efficacy trial. The analytic emphasis is on estimating recruitment, retention, outcome completion, safety, tolerability, usability, acceptance, adherence, and variability in cognitive and delirium-related measurements. Reporting will be informed by SPIRIT (Standard Protocol Items: Recommendations for Interventional Trials) guidelines (Checklist 1), the CONSORT (Consolidated Standards of Reporting Trials) extension for pilot and feasibility trials (Multimedia Appendix 1), and CONSORT-EHEALTH (Consolidated Standards of Reporting Trials of Electronic and Mobile Health Applications and Online Telehealth) guidance for digital health interventions.

### Participants

Figure 1 shows the study schema, and Table 1 summarizes the study-scheduled activities. Eligible participants are adults aged 65 years or older who will be undergoing elective or emergency major surgery with expected postoperative ICU admission for approximately 3 days. Participants must have mild-to-moderate cognitive impairment, defined by a Montreal Cognitive Assessment (MOCA) score of 15 to 24 [34]. This range identifies patients at increased risk of delirium who may still be able to interact with a low-movement cognitive intervention.

**Figure 1.**
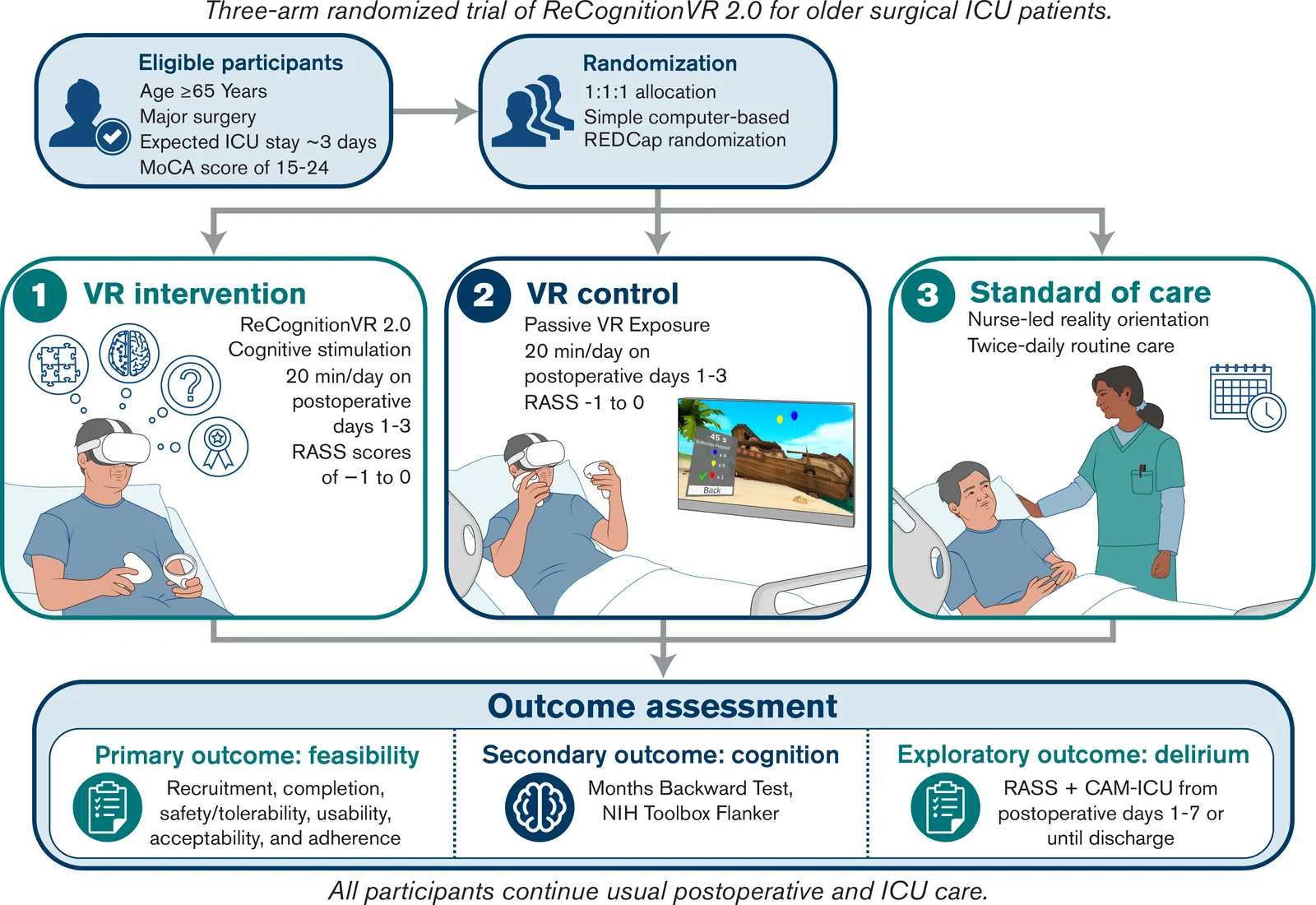
Study schema. Eligible older adults with mild-to-moderate cognitive impairment undergoing major surgery and expected ICU admission are randomized by simple computer-based REDCap randomization in a 1:1:1 ratio to ReCognitionVR 2.0 cognitive stimulation, passive VR control, or standard-of-care nurse-led reality orientation. CAM-ICU: Confusion Assessment Method for the Intensive Care Unit; ICU: intensive care unit; MoCA: Montreal Cognitive Assessment; NIH: National Institutes of Health; RASS: Richmond Agitation-Sedation Scale; VR: virtual reality.

**Table 1.** Study schedule: activities and events.

Procedures	Preoperative visit	Surgery	Postoperative day 1	Postoperative day 2	Postoperative day 3	Postoperative days 4‐7	Hospital discharge	Study personnel
Study day	−30 to 0^a^	0	1	2	3	4‐7	8+^b^	
Visit number	Screening		1	2	3	4‐7	8	
Prescreening and informed consent	✓							CRC^c^/PI^d^
Montreal Cognitive Assessment	✓							CRC
Concomitant medication review	✓							CRC
Baseline medical and surgical history	✓							CRC
Physical examination (including height and weight)	✓							CRC/PI
Richmond Agitation-Sedation Scale	✓		✓	✓	✓	✓	✓	CRC
Randomization			✓					CRC
Administration of assigned study intervention			✓	✓	✓			CRC
VR^e^ sessions (VR intervention and VR control): one 20-min session per day on postoperative days 1‐3								
Vital signs (BP^f^, HR^g^, RR^h^, and SpO_2_^i^) and numeric pain score			✓	✓	✓			CRC
Adverse event review and evaluation			✓	✓	✓	✓		PI
System Usability Scale							✓	CRC/PI
Behavioral Intention Scale							✓	CRC/PI
Nurse-led reality orientation (nurse control): twice daily as part of routine care								
Vital signs and pain scores (morning, before orientation, and evening)			✓	✓	✓			Nurse
Tests administered to all study arms								
Columbia-Suicide Severity Rating Scale Screen	✓							CRC/PI
Months Backward Test			✓		✓			CRC/PI
NIH^j^ Flanker Inhibitory Control and Attention Test			✓		✓			CRC
Confusion Assessment Method for the ICU^k^	✓		✓	✓	✓	✓	✓	OA^l^^,^^m^

a Participants undergoing emergency surgery may be enrolled on the day of surgery before anesthesia.

b Hospital discharge may occur before or after study day 8, depending on recovery.

c CRC: clinical research coordinator.

d PI: principal investigator or medical coinvestigator.

e VR: virtual reality.

f BP: blood pressure.

g HR: heart rate.

h RR: respiratory rate.

i SpO_2_: peripheral oxygen saturation.

j NIH: National Institutes of Health.

k ICU: intensive care unit.

l OA: outcome assessor.

m Outcome assessors will be blinded to treatment assignment.

Key exclusion criteria include preexisting intubation and invasive mechanical ventilation, severe mental illness, admission for drug overdose, severe vertigo, history of severe postoperative nausea and vomiting, hearing or visual impairment that would preclude VR use, severe dementia defined by a MoCA score below 15, active delirium at screening, and active suicidal ideation or behavior. Participants who require prolonged postoperative intubation for more than 24 hours will be considered screen failures after surgery.

### Recruitment, Screening, Consent, and Assent

Potential participants will be identified through surgeon clinics, preoperative workflows, and hospital admission lists. Screening occurs 1 to 30 days before surgery, with final eligibility confirmed before enrollment. Written informed consent will be obtained from the participant or a legally authorized representative (LAR) before study-specific procedures are performed.

Because the intervention is preventive, patients with active delirium at screening will be excluded. Suicidality screening will include medical record review and the Columbia-Suicide Severity Rating Scale Screen [35]. Patients with evidence of active suicidal ideation or behavior will not be enrolled and will be referred for clinical evaluation according to institutional policy.

The study population includes older adults with mild-to-moderate cognitive impairment; therefore, decisional capacity will be assessed when a LAR is not available. Capacity assessment will address the participant’s ability to understand the study purpose and procedures, appreciate personal implications of participation, reason about alternatives and consequences, and communicate a voluntary and consistent choice.

For participants who lack full decisional capacity, written informed consent will be obtained from a LAR before enrollment. Assent will be sought from participants who retain sufficient ability to understand the study at a basic level. Study personnel will use simplified language to explain the study’s purpose, procedures, the voluntary nature of participation, and the right to stop. Behavioral dissent, verbal refusal, distress, or resistance will be respected regardless of LAR consent. Assent or dissent will be documented in the study record.

A meeting review after enrollment began identified the need for standardized communication regarding research MoCA results. Research MoCA scores will not be used to make a clinical diagnosis. If a participant requests their result and the institutional review board permits disclosure, the result will be accompanied by written language explaining that preoperative anxiety, illness, and the testing context can affect performance, and that follow-up with primary care or neurology may be appropriate if concerns persist after recovery.

### Randomization and Blinding

After eligibility confirmation and baseline assessment, participants will be randomized in a 1:1:1 ratio using simple computer-based randomization implemented through the REDCap randomization module [36]. Simple randomization was selected because the trial is a small pilot study in which complex stratification could increase operational burden and create sparsely populated strata. Participants and intervention staff cannot be blinded to VR exposure. Participants will be blinded to the study hypothesis. Outcome assessors administering cognitive and delirium measures will remain blinded to group assignment. Data analysts will be blinded to allocation to the extent feasible, recognizing that usability data apply only to participants exposed to VR.

### Development of ReCognitionVR 2.0

Figure 2 shows the iterative development model from the prototype to ReCognitionVR 2.0, representing a major redevelopment of an earlier ReCognitionVR prototype evaluated in preliminary feasibility studies [32,33]. The upgrade was guided by human-centered design principles, prior bedside feasibility experience, staff feedback, engineering review, and ICU workflow constraints. The goal was to reduce operational complexity, improve intervention fidelity, and increase suitability for older hospitalized and critically ill patients.

Key refinements included a simplified launch interface, reduced setup burden, improved controller usability, decreased dependence on real-time headset casting, and enhanced device-generated performance capture. In this context, casting refers to wirelessly mirroring the VR headset display to an external device, such as a tablet or bedside monitor, so staff can observe what the participant sees during the session. Although casting can support staff observation and troubleshooting, it may be affected by variability across hospital networks, device pairing challenges, audio or visual lag, and additional workflow demands. Therefore, ReCognitionVR 2.0 prioritized direct in-app data capture and dashboard-based monitoring to reduce reliance on continuous live mirroring during bedside implementation.

**Figure 2.**
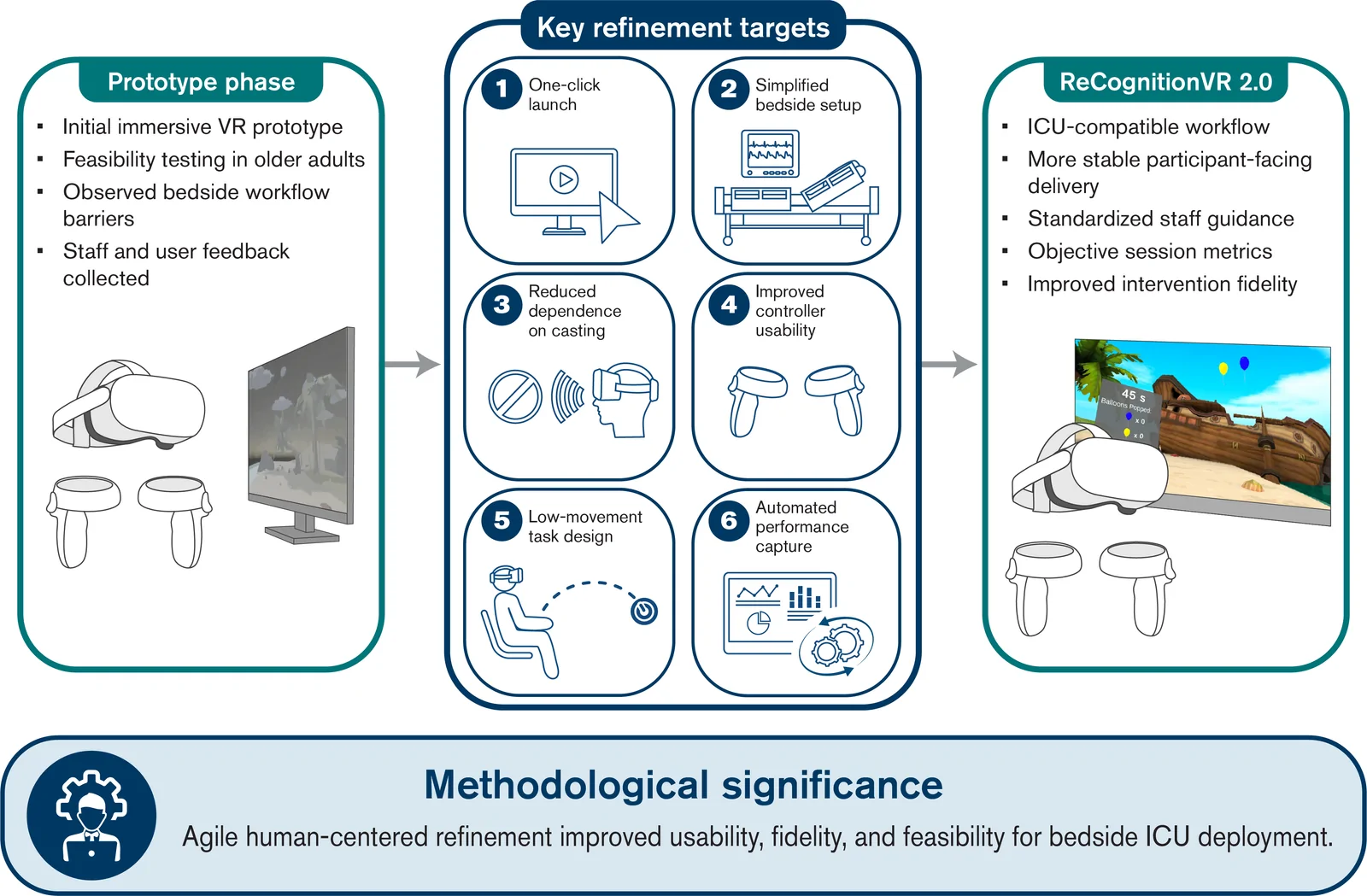
Iterative development model from prototype to ReCognitionVR 2.0. Prototype feasibility findings and steering committee feedback informed upgrades focused on simplified launch, reduced setup burden, improved controller usability, reduced dependence on casting, low-movement task design, automated performance capture, standardized staff guidance, and ICU-compatible workflow integration. ICU: intensive care unit; VR: virtual reality.

A central methodological advance is the automated capture of game performance data. The platform records game difficulty, head movement, controller movement, and movement of virtual objects during each game, from which performance-indicative metrics may be derived.

The engineering review documented that headset and controller position and rotation data can be captured at approximately 60 Hz, with time-stamped game events such as balloon spawn, first visibility, hover, user pop, and auto-pop. These data reduce reliance on manual recording and permit exploratory derivation of engagement, attention, latency, inattention, completion efficiency, and performance-stability metrics.

The meeting-based usability review also identified future software development priorities that are not protocol-changing interventions for the current trial. These include more explicit virtual-nurse instructions for easy, medium, hard, and survival modes; fixed forward-facing interaction zones; reduced extraneous cognitive load; and potential future task modules targeting selective attention, inhibition, working memory, rule switching, processing speed, and error monitoring. These items will inform future development of ReCognitionVR 3.0 and a subsequent R01-stage protocol.

### Intervention Arms

#### ReCognitionVR 2.0–Based Cognitive Stimulation Arm

Participants randomized to the intervention arm will receive brief video training on how to use a VR headset before engaging with ReCognitionVR 2.0 [37]. Sessions will be delivered at the bedside on postoperative days 1 through 3, once daily for up to 20 minutes, when the participant is calm and alert, defined by a Richmond Agitation-Sedation Scale (RASS) score of −1 to 0 [38].

Figure 3 shows the ReCognitionVR 2.0 intervention workflow. Each session begins with a readiness assessment, symptom screening, vital sign measurement, and headset placement. The immersive environment is intentionally calm and low-stimulus. A virtual nurse avatar provides orientation to time, place, situation, and the hospital environment. Participants then complete low-movement cognitive games, including balloon-popping tasks that require attention, target selection, visuomotor coordination, and task persistence. Difficulty may increase according to performance.

**Figure 3.**
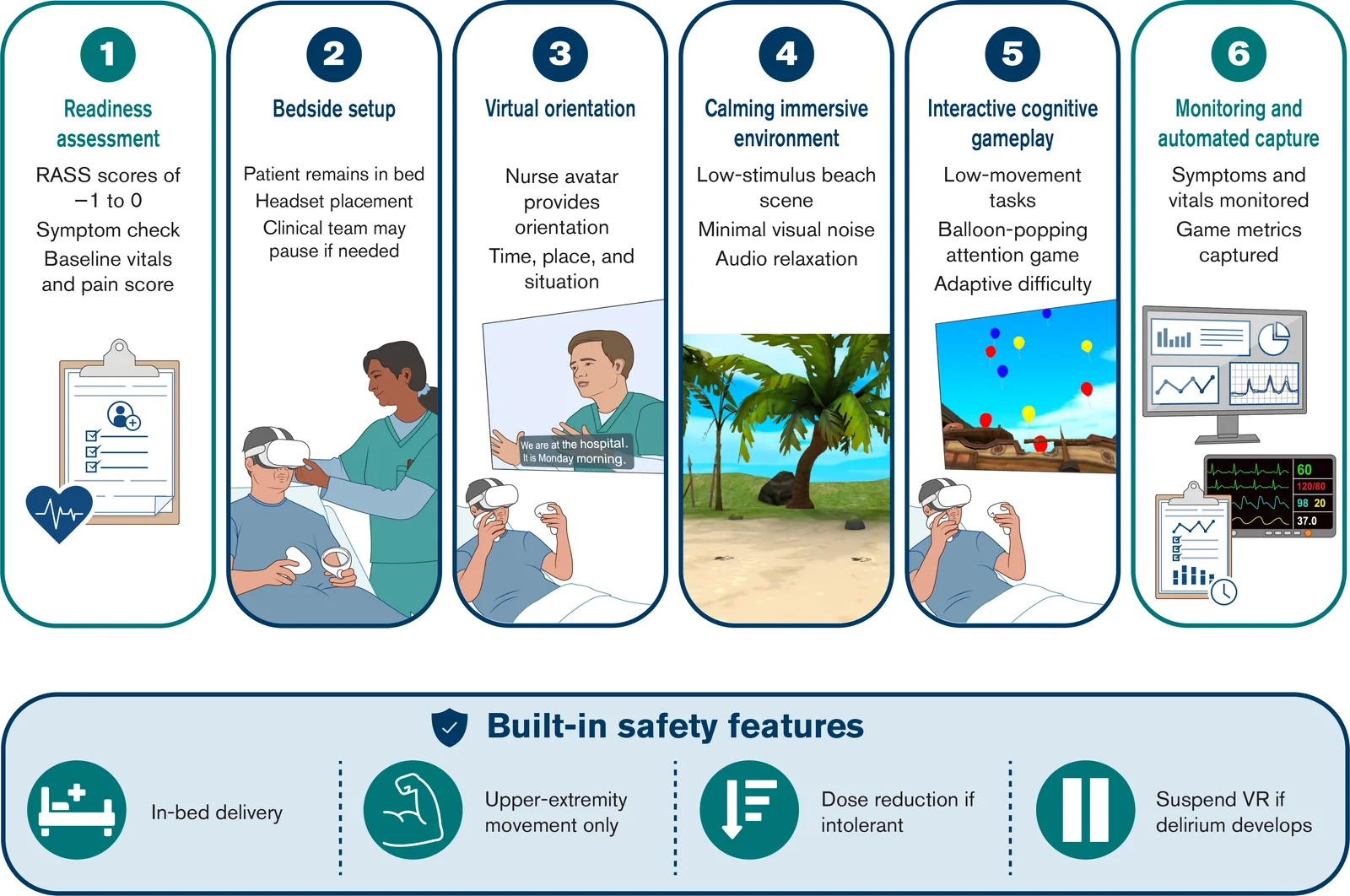
ReCognitionVR 2.0 intervention workflow. The bedside workflow includes readiness assessment, oxygen/readiness checklist, headset placement, orientation via a virtual nurse avatar, a calming, immersive environment, low-movement interactive cognitive gameplay, symptom and vital sign monitoring, and automated performance capture. RASS: Richmond Agitation-Sedation Scale; VR: virtual reality.

The ReCognitionVR 2.0 platform comprises a Meta Quest 3 VR headset (Meta Platforms Inc), 2 Meta Quest Touch Plus controllers, and an iPad-based monitoring interface. The software was developed in Unity 6 (Unity Technologies) and renders an immersive 3-dimensional beach environment. Participants remain in bed and use low-amplitude upper-extremity movements to interact with virtual objects.

#### Passive VR Control Arm

Participants randomized to the passive VR control arm will use the same headset platform but will view noninteractive VR content without active cognitive gameplay or adaptive task demands. The comparator controls headset exposure, novelty, visual and auditory immersion, staff presence, and time spent in a virtual environment. Steering committee review confirmed that the current passive VR control remains appropriate because early participants tolerated the control condition, remained in the headset for approximately 20 minutes, and did not decline to continue participating on subsequent postoperative days.

#### Standard-of-Care Nurse-Led Reality Orientation Arm

Participants randomized to the nurse-led control arm will receive standard-of-care reality orientation focused on time, place, person, and situation. Orientation will be delivered by clinical nursing staff as part of routine care, generally at the beginning and end of nursing shifts. Participants in all arms will continue to receive routine delirium-prevention and ICU care as clinically indicated.

#### Safety Monitoring and Intervention Modification

All VR sessions will occur while participants remain in bed in a supine or semirecumbent position. The ICU team retains authority to delay, pause, or terminate sessions if clinical care takes priority or the participant becomes clinically unstable. The VR system does not electronically interface with ICU medical devices and will be positioned to avoid vascular access, monitoring cables, oxygen devices, infusion pumps, and other bedside equipment.

Vital signs and pain scores using the numeric rating pain scale will be collected before, approximately 10 minutes into, and after each VR session [39]. Sessions will be stopped for unresolved adverse symptoms, participant request, RASS score outside the permitted range, unsafe movement, clinical care interruption, or a greater than 20% increase from baseline in heart rate, blood pressure, or respiratory rate. VR-related symptoms of interest include dizziness, nausea, eye strain, visual discomfort, headache, anxiety, fatigue, and cybersickness symptoms [40-42].

If a session is stopped because of intolerance or abnormal vital signs, subsequent sessions may be reduced from 20 minutes to 10 minutes or divided into two 10-minute sessions if clinically appropriate. If a participant develops active delirium, defined as any positive Confusion Assessment Method for the ICU (CAM-ICU) assessment [43], or no longer meets alertness criteria, VR sessions will be suspended. These participants will continue to receive standard-of-care delirium management and will remain in follow-up under an intention-to-treat framework.

Early implementation review identified one oxygen-connection near miss in which postoperative oxygen saturation decreased after the participant had not been reconnected to oxygen following respiratory treatment. The event did not meet the protocol adverse event (AE) threshold, but it led to a preintervention oxygen/readiness checklist. Before headset placement, the research team will confirm that the participant has returned to baseline respiratory-support status, including oxygen tubing or cannula connection, wall oxygen or cylinder status, and baseline oxygen saturation.

### Outcomes

The primary outcome is the feasibility of the trial and intervention. Feasibility will be assessed through recruitment rate, outcome data completion, safety and tolerability, usability, acceptance, and adherence. Recruitment will be summarized by the numbers approached, agreeing, and consenting, as well as by monthly accrual. Outcome data completion will be quantified using 2 prespecified denominators. The primary completion metric will be the number of completed protocol-scheduled assessments divided by the number of assessments that became due from randomization through the end of follow-up. An assessment will be considered due if its scheduled time point occurs before the earliest of hospital discharge, death, withdrawal of consent, or study discontinuation; assessments that are not due because follow-up has ended will be excluded from the denominator. Assessments that are due but not completed because of clinical instability, participant refusal, competing clinical care, logistical barriers, or inability to complete the assessment will remain in the primary denominator and will be categorized by reason for noncompletion. As a secondary descriptive metric, evaluable-assessment completion will be calculated among due assessments for which the participant met prespecified assessment-specific eligibility criteria (eg, adequate arousal and ability to respond for cognitive testing, and VR exposure for usability and acceptance measures). Safety and tolerability will be summarized using AE rates, session interruptions, and short-interval changes in vital signs. Table 2 explains the overall outcome and measurement strategy, and Table 3 summarizes the feasibility benchmarks for progression planning.

**Table 2.** Outcomes and measurement strategy.

Domain	Outcome	Measurement	Time frame
Feasibility	Recruitment	Number approached, agreed, and consented; monthly accrual	Screening through end of enrollment
Feasibility	Outcome completion	Primary: completed due protocol-scheduled assessments divided by all assessments due before the end of follow-up. Secondary: completed due assessments divided by due assessments for which the participant met assessment-specific eligibility criteria	Through hospital discharge
Safety/tolerability	Adverse events and vital sign changes	Solicited and unsolicited adverse events; BP^a^, HR^b^, RR^c^, SpO_2_^d^, pain score; session interruptions	Postoperative days 1‐3; adverse event follow-up through postoperative day 7 or discharge
Usability	System Usability Scale	Mean score among VR^e^-exposed participants	Hospital discharge
Acceptance	Behavioral Intention Scale	Four-item behavioral intention measure	Hospital discharge
Adherence	Session completion	Completion of at least 2 protocol-defined VR sessions, allowing dose modification	Postoperative days 1‐3
Cognition	Attention and executive function	Months Backward Test; NIH^f^ Toolbox Flanker Inhibitory Control and Attention Test	Postoperative day 1 and postoperative day 3 or discharge if earlier
Digital performance	Engagement and task performance	Game accuracy, difficulty level, d′^g^, reaction time, completion efficiency, task progression, head-gaze measures, session duration	Each ReCognitionVR session
Clinical exploratory	Postoperative delirium incidence	RASS^h^ followed by CAM-ICU^i^; at least 1 CAM-ICU-positive episode	Postoperative days 1‐7 or discharge

a BP: blood pressure.

b HR: heart rate.

c RR: respiratory rate.

d SpO_2_: oxygen saturation.

e VR: virtual reality.

f NIH: National Institutes of Health.

g d′: d-prime.

h RASS: Richmond Agitation-Sedation Scale.

i CAM-ICU: Confusion Assessment Method for the Intensive Care Unit.

**Table 3.** Feasibility benchmarks for progression planning.

Metric	Target	Interpretive use
Recruitment rate	4‐5 participants per month	Determines whether the enrollment workflow is practicable
Consent rate	At least 60% of approached eligible patients	Assesses acceptability of study participation
Outcome completion	At least 80% primary completion of due protocol-scheduled assessments	Evaluates tolerability and data completeness
VR^a^ adherence	At least 75% complete at least 2 VR sessions	Assesses intervention deliverability
Usability	Mean System Usability Scale score >70	Evaluates patient-facing usability
Acceptance	At least 70% moderate-to-high behavioral intention	Evaluates perceived acceptability and willingness to use
Data quality	Missing data <5% per form; query resolution <7 days	Assesses readiness for larger-trial infrastructure
Safety	No concerning clustering of serious or moderate/severe VR-related adverse events	Supports progression to larger feasibility or efficacy testing

a VR: virtual reality.

Usability will be assessed using the System Usability Scale among VR-exposed participants [44]. Acceptance will be assessed using a 4-item Behavioral Intention Scale at hospital discharge [45,46]. Adherence will be defined as completion of at least 2 VR sessions in accordance with the assigned VR protocol, with dose modification when clinically indicated.

Secondary outcomes estimate preliminary effects on attention and executive function. The Months Backward Test will be assessed using both a pass/fail threshold and the number of correct months recited before the first error [47]. The NIH Toolbox Flanker Inhibitory Control and Attention Test will provide measures of accuracy and reaction time [48].

Device-generated performance metrics will be treated as exploratory digital end points as described in Table 4. Prespecified and candidate metrics by difficulty level include session duration, sensitivity to signal presence, reaction-time summaries, idle time as a potential marker of inattention, commitment percentage, head-gaze-derived attention stability, and engagement trajectories across epochs (beginning, middle, and end of the session).

**Table 4.** Candidate virtual reality–derived digital performance metrics.^a^

Metric category	Candidate variable	Interpretation
Sensitivity	True positives, false positives, true negatives, false negatives, d′^b^	Overall sensitivity to responding to signal-present stimuli while avoiding false alarms
Latency	Time from stimulus visibility to first response; reaction-time summaries	Processing speed and response initiation
Inattention	Idle time; prolonged latency; no response to eligible visible targets	Possible attentional lapse or fatigue
Commitment	Task completion percentage	Sustained interaction with the assigned task
Attention stability	Head-gaze-derived stability ratio	Exploratory proxy requiring empirical validation
Engagement	Change in reaction time over a session	Increasing effort in response to increasing difficulty
Context annotation	Game-level comments for distraction, early quitting, assistance, or technical issues	Human-factors interpretation and data-quality review

a Candidate metrics are exploratory and will require empirical validation before use as efficacy end points.

b d′: d-prime.

The exploratory clinical outcome is the incidence of postoperative delirium. Delirium will be assessed using the CAM-ICU after arousal is confirmed with the RASS [38,43]. Delirium incidence is defined as at least 1 CAM-ICU-positive episode from postoperative days 1 through 7 or until hospital discharge, whichever occurs first.

### Schedule of Assessments

Screening assessments include informed consent or LAR consent, assent when applicable, medical and surgical history, the MoCA, the RASS, the CAM-ICU, physical examination a review of concomitant medications, and suicidality screening. On postoperative day 1, participants will undergo baseline cognitive assessment using the Months Backward Test and the NIH Toolbox Flanker Inhibitory Control and Attention Test, then be randomized and, if clinically eligible, receive the first assigned intervention. VR sessions and safety assessments will occur on postoperative days 1 through 3. Delirium assessments will continue through postoperative days 1 to 7 and at hospital discharge. Usability and acceptance measures will be collected at discharge (Table 1).

### Data Collection and Management

Study data will be entered into REDCap using standardized case report forms [36]. Data elements include recruitment and consent metrics, eligibility criteria, intervention delivery, adherence, vital signs, AEs, cognitive assessments, delirium assessments, usability surveys, acceptance measures, and device-generated VR performance metrics. Identifiable data will be stored separately from study data using unique participant identifiers. Data quality will be supported by staff training, standard operating procedures, source data verification, REDCap query resolution, protocol deviation tracking, and periodic review as part of the study quality-control process. Blinded outcome assessors will receive standardized training in cognitive and delirium assessments. Case report forms and intervention logs will be reviewed for completeness, consistency, and timeliness.

### Sample Size

The planned sample size is 90 participants, with 30 participants per arm. This sample size was selected to estimate feasibility metrics with clinically useful precision, generate preliminary estimates of variability and effect size for cognitive outcomes, evaluate safety and tolerability signals, and inform design assumptions for a future efficacy trial. No formal hypothesis-testing power calculation was performed because the trial is designed primarily to estimate feasibility.

### Statistical Analysis

Analyses will follow the intention-to-treat principle and primarily focus on feasibility and the estimation of intervention effects rather than on formal hypothesis testing. Feasibility outcomes will be summarized descriptively using counts, percentages, means with SDs, medians with IQRs for continuous variables, along with 95% CIs, as appropriate. Feasibility benchmarks will be interpreted as progression criteria rather than formal hypothesis tests.

For cognitive outcomes, between-group differences at follow-up will be estimated using effect sizes and 95% CIs. Linear mixed-effects models may be used to account for repeated measurements and within-participant correlation, including a random intercept for participant. Models will adjust for baseline cognitive performance and, where sample size permits, a limited set of clinically relevant covariates to avoid overfitting.

Device-generated performance metrics will be summarized descriptively and explored as indicators of intervention engagement, fidelity, and tolerance. Where appropriate, these metrics may also be examined in relation to clinical outcomes in exploratory analyses.

For delirium incidence, odds ratios and 95% CIs will be estimated using logistic regression if event counts permit. Candidate covariates include age, baseline cognitive function, sex, race or ethnicity, surgery type, anesthesia duration, comorbidity burden, and other clinically relevant delirium risk factors. If event counts are sparse, penalized or exact logistic regression methods may be considered, or analyses may be limited to descriptive comparisons.

Missing data will be described by study arm and outcome domain. Sensitivity analyses may examine worst-case assumptions for binary outcomes and model-based approaches for continuous outcomes (eg, mixed-effects models using maximum likelihood), which are valid under missing-at-random assumptions.

A protocol deviation involving the omission of the instructional video for one participant in a VR intervention will be flagged in the analytic dataset. If this participant’s VR performance metrics differ materially from those of other participants, sensitivity analyses will assess the impact of including versus excluding these data in exploratory digital performance analyses, subject to IRB and analytic review.

### Ethical Considerations

The study will be conducted under Houston Methodist Institutional Review Board (IRB) approval and in accordance with Good Clinical Practice, the Declaration of Helsinki, institutional human-subjects protection policies, and applicable regulatory requirements. The modified protocol is version 2, dated April 13, 2026. The protocol identifies the study as a nonsignificant risk device study under 21 CFR 812.3(m) with FDA IDE G260027. Deidentified data sharing will comply with institutional policy, IRB requirements, and National Institutes of Health data-sharing expectations.

### Dissemination

Results will be disseminated through peer-reviewed publication and scientific presentation. Authorship will follow the International Committee of Medical Journal Editors criteria.

## Results

This study was funded in September 2025. Enrollment began on April 24, 2026, after safety officer approval and programmatic authorization to proceed. During the early recruitment period, 59 patients were prescreened through chart review without consent. Of these, 24 were considered potentially eligible and were approached for participation. Six patients were subsequently excluded: 1 because of prolonged postoperative intubation exceeding 24 hours, 2 because their MoCA scores were greater than 24, 2 because they did not require postoperative ICU admission, and 1 because of a history of postoperative nausea and vomiting. As of July 29, 2026, 18 participants had been enrolled and allocated equally across the 3 study arms: 6 to the ReCognitionVR intervention, 6 to the passive VR control, and 6 to standard care. The CONSORT flow diagram (Figure 4) summarizes recruitment, screening, enrollment, allocation, intervention delivery, follow-up, and analysis status. Based on the current recruitment rate, sustained screening volume, and similar eligibility patterns, we project enrollment of the planned 90 participants will be completed by mid-2027.

**Figure 4.**
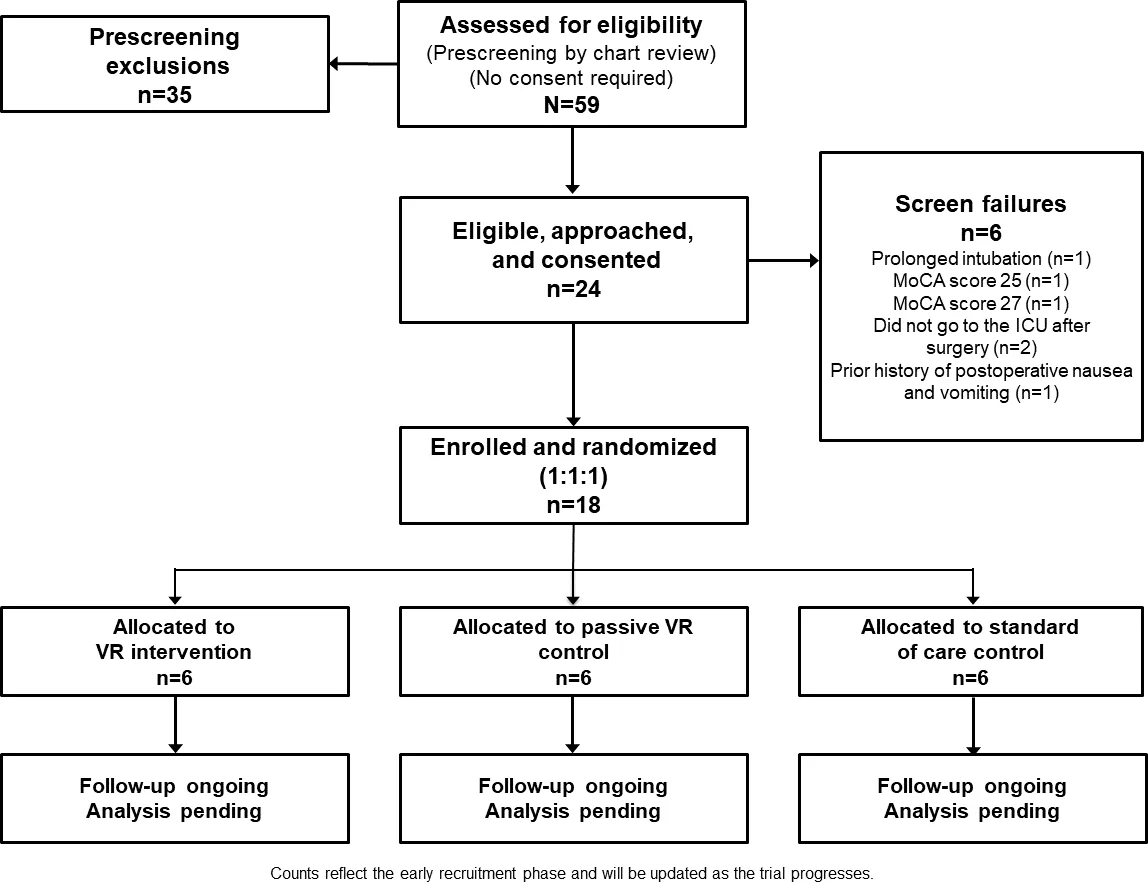
CONSORT (Consolidated Standards of Reporting Trials) flow diagram. Early recruitment phase (April 24 to July 29, 2026). MoCA: Montreal Cognitive Assessment; VR: virtual reality

Early operational review generated implementation and safety information without altering the approved intervention. The first participant assigned to the ReCognitionVR arm completed shortened sessions without physiologic instability. Frustration with game performance and postoperative hand tremors were documented but were not classified as serious VR-related AEs. One minor AE consisting of transient blurred vision resolved spontaneously within approximately 5 minutes and required no treatment. We documented a missed instructional video as a minor protocol deviation and flagged it for analytic review. This represented documentation and planned analytic handling of a deviation rather than a modification of the intervention.

The IRB-approved protocol prespecified automated capture of device- and game-generated data, collection of exploratory digital performance measures, and routine tracking of protocol deviations. Prespecified VR performance measures included game accuracy in the easy, medium, and hard modes (percentage of balloons popped) and survival-mode completion time (mean duration participants maintained 100% accuracy across 3 attempts). After enrolling the first participant in the ReCognitionVR arm, we refined the operational definitions and planned reporting of these exploratory VR-derived outcomes to improve clarity and consistency; the underlying data-collection procedures remained unchanged. Current VR performance outcomes include survival-mode completion time, d′ (“d-prime”), minimum, maximum, and mean reaction times, idle time, commitment percentage, attention stability ratio, and engagement score. We are still working to derive some of the exploratory VR performance metrics.

Following an oxygen-connection near miss, a preintervention oxygen/readiness checklist was introduced to confirm that participants had returned to their baseline respiratory-support status before headset placement. The Study Quality Control Committee reviewed the checklist and determined that it could be implemented as an operational quality-control and safety safeguard without requiring a protocol amendment. These refinements did not affect participant eligibility, treatment allocation, the cognitive intervention, study outcomes, or the data-collection schedule. Results are expected to be published in Winter 2027.

## Discussion

### Principal Considerations

This protocol describes a randomized pilot trial of ReCognitionVR 2.0, an immersive VR-based cognitive stimulation intervention designed for older surgical ICU patients with mild-to-moderate cognitive impairment. The study addresses a major implementation gap in delirium prevention: the need for scalable, engaging, standardized, and monitorable nonpharmacologic interventions that can be delivered within high-acuity postoperative care [15,49].

The principal innovation is the integration of orientation, environmental modulation, low-movement interactive tasks, adaptive difficulty, and device-generated performance capture into a single bedside workflow. The transition from the original prototype to ReCognitionVR 2.0 is methodologically important because it reflects iterative refinement informed by feasibility data, workflow barriers, and human factors considerations. The revised platform reduces setup complexity, limits reliance on network-dependent casting, simplifies staff-facing operation, and provides objective performance data for future end point development.

The protocol treats human-factors outcomes as central feasibility outcomes. Usability, acceptance, adherence, session interruption, task completion, physiologic tolerance, and staff workflow compatibility are essential when introducing technology into critical care. An intervention that is cognitively plausible but operationally fragile is unlikely to be scalable. By measuring these domains prospectively, the trial will clarify whether immersive VR cognitive stimulation can be implemented with sufficient fidelity in a vulnerable postoperative ICU population.

The passive VR control arm strengthens the design by distinguishing active cognitive stimulation from nonspecific effects of headset use, novelty, immersion, staff attention, and time in a virtual environment. This distinction is important for future interpretation of efficacy. If cognitive or delirium-related signals emerge, comparison with passive VR exposure will help determine whether the signal is attributable to active task engagement rather than VR exposure alone.

### Safety and Implementation Considerations

The safety framework is intentionally conservative. Older postoperative ICU patients with cognitive impairment are susceptible to postoperative pain, dizziness, nausea, visual discomfort, hemodynamic instability, fatigue, delirium, and clinical interruptions [50-56]. The protocol mitigates these risks by requiring a calm and alert state before each session, conducting all sessions in bed, restricting tasks to low-amplitude upper-extremity movement, monitoring vital signs before, during, and after sessions, allowing dose modification, and suspending VR exposure if delirium develops. Early experience further underscores the importance of readiness checks, particularly oxygen-connection verification and confirmation of clinical stability before headset placement.

### Limitations

The study has limitations. It is a single-center study and not powered to test clinical efficacy. Participants and intervention staff cannot be blinded to VR exposure. Clinical heterogeneity across surgical populations may influence cognitive recovery and the risk of delirium. Simple randomization may lead to baseline imbalances across arms, although this is acceptable for a feasibility study and will be considered in interpretation. Several VR-derived measures, including head gaze–based attention stability and engagement trajectories, are being explored and require empirical validation before they can be used as efficacy end points. Finally, delirium incidence is included as an exploratory outcome and should be interpreted as hypothesis-generating.

### Future Directions

This study is positioned to generate essential information for the next phase of investigation. It will determine whether older surgical ICU patients with cognitive impairment can be recruited, randomized, exposed to immersive VR, monitored safely, and assessed with sufficient completeness. It will also provide estimates of intervention tolerance, device usability, adherence, variability in cognitive outcomes, feasibility of delirium ascertainment, and operational barriers. Meeting-based feedback has already identified future software development priorities, including more autonomous, avatar-guided instructions, additional cognitive domains, and enhanced digital phenotyping. These data will inform optimization of ReCognitionVR 3.0 and design of a future adequately powered multicenter trial.

### Conclusions

This randomized pilot trial will evaluate the feasibility, usability, safety, acceptance, adherence, and preliminary cognitive and delirium-related outcomes of ReCognitionVR 2.0 in older surgical ICU patients with mild-to-moderate cognitive impairment. By combining immersive cognitive stimulation, bedside orientation, conservative safety monitoring, and device-generated performance metrics, the trial will provide critical methodological data for developing scalable VR-enabled delirium-prevention strategies in critical care.

## Supplementary material

10.2196/105624 Multimedia Appendix 1 Approved protocol synopsis.

10.2196/105624 Checklist 1 SPIRIT checklist.

## Abbreviations

AE: adverse event
CAM-ICU: Confusion Assessment Method for the Intensive Care Unit
CONSORT: Consolidated Standards of Reporting Trials
CONSORT-EHEALTH: Consolidated Standards of Reporting Trials of Electronic and Mobile Health Applications and Online Telehealth
ICU: intensive care unit
IRB: institutional review board
LAR: legally authorized representative
MoCA: Montreal Cognitive Assessment
RASS: Richmond Agitation-Sedation Scale
SPIRIT: Standard Protocol Items: Recommendations for Interventional Trials
VR: virtual reality
