# Supplementary material for: Immersive Virtual Reality Cognitive Stimulation for Delirium Prevention in Older Surgical Intensive Care Unit Patients With Cognitive Impairment: Protocol for a 3-Arm Randomized Pilot Trial
Source: JMIR Res Protoc. 2026 Nov 5;15:e105624. doi: 10.2196/105624 (PMC13740225; doi:10.2196/105624)
Supplement: Supplementary file 1 [file resprot-v15-e105624-s001.pdf]

Multimedia Appendix 2. Approved Protocol Synopsis

ReCognitionVR 2.0 randomized pilot trial

Purpose. This appendix provides a publication-facing synopsis of the approved modified protocol. It is intended for upload to JMIR when institutional policy does not permit public dissemination of the full IRB protocol document.

Protocol element	Synopsis
Title	Virtual Reality-Based Cognitive Stimulation Games for Enhancing Attention and Preventing Delirium in Older Surgical Patients with Cognitive Impairment: A Randomized Controlled Pilot Trial.
Short title	ReCognitionVR 2.0 for delirium prevention in older surgical ICU patients.
Protocol and regulatory identifiers	Houston Methodist protocol PRO00041199; modified protocol version 2 dated April 13, 2026; FDA IDE G260027; nonsignificant risk device study under 21 CFR 812.3(m).
Funding	National Institute on Aging, 1K76AG088424-01A1.
Study design	Single-center, three-arm, parallel-group randomized pilot trial with 1:1:1 allocation to VR-based cognitive stimulation, passive VR control, or standard-of-care nurse-led reality orientation.
Setting	Surgical intensive care units at Houston Methodist Hospital, Houston, Texas.
Population	Adults aged 65 years or older undergoing elective or emergency major surgery with expected postoperative ICU admission for approximately three days and mild-to-moderate cognitive impairment defined by MoCA score 15 to 24.
Key exclusions	Pre-existing intubation and invasive ventilation; severe mental illness; admission for drug overdose; severe vertigo; severe postoperative nausea/vomiting history; hearing/visual impairment precluding VR use; MoCA <15; active delirium at screening; active suicidal ideation or behavior; prolonged postoperative intubation >24 hours.
Consent, capacity, and assent	Written informed consent will be obtained from participants or legally authorized representatives. Capacity will be assessed when needed. Assent will be sought from participants with sufficient understanding; behavioral dissent or refusal will be respected even when LAR consent is present.
Randomization and blinding	Participants will be randomized using a simple computer-based REDCap randomization after the baseline assessment. Participants and intervention staff cannot be blinded to VR exposure; outcome assessors will remain blinded to group assignment.
Intervention arm	ReCognitionVR 2.0-based cognitive stimulation delivered at the bedside once daily for up to 20 minutes on postoperative days 1 through 3 when RASS is -1 to 0. The intervention includes virtual nurse orientation, calming immersive environment, low-movement cognitive gameplay, adaptive difficulty, and automated performance capture.
Passive VR control arm	Participants use the same VR platform for passive noninteractive exposure without cognitive gameplay or adaptive task demands, controlling for headset exposure, immersion, novelty, staff presence, and time in VR.
Nurse-led control arm	Standard-of-care reality orientation focused on time, place, person, and situation, delivered by clinical nursing staff as part of routine care.
Primary feasibility outcomes	Recruitment rate, outcome data completion, safety/tolerability, usability, acceptance, and adherence.
Secondary cognitive outcomes	Months Backward Test and NIH Toolbox Flanker Inhibitory Control and Attention Test; device-generated performance metrics including accuracy, difficulty, completion time, task progression, and session duration.
Exploratory clinical outcome	Postoperative delirium incidence is defined as at least one CAM-ICU-positive episode from postoperative days 1 through 7 or until hospital discharge, whichever occurs first.
Safety monitoring	VR sessions occur in bed, with vital signs and pain scores assessed before, approximately 10 minutes into, and after each session. Sessions stop for unresolved symptoms, participant request, unsafe movement, RASS outside -1 to 0, clinical interruption, or >20% increase from baseline in heart rate, blood pressure, or respiratory rate. VR is suspended if delirium develops.
Sample size	N=90 participants, 30 per arm, selected to estimate feasibility metrics, safety/tolerability, variability, and effect-size parameters for a future trial. No formal efficacy power calculation is used.
Analysis plan	Feasibility metrics will be summarized descriptively with confidence intervals. Cognitive outcomes will be analyzed using effect sizes and, when feasible, mixed-effects models. Delirium incidence will be explored using odds ratios and logistic regression if event counts permit. Analyses will follow the intention-to-treat principle.
Data management	Study data will be entered into REDCap using standardized case report forms. Data quality will be supported by training, source data verification, query resolution, protocol deviation tracking, and quality-control review.
Ethics and dissemination	The study will be conducted under the oversight of the Houston Methodist Institutional Review Board and in accordance with Good Clinical Practice, the Declaration of Helsinki, institutional human subjects protections, and applicable regulatory requirements. Results will be disseminated through peer-reviewed publication and scientific presentation.

Document control note. Before submission, verify whether the full IRB-approved protocol may be uploaded instead of, or in addition to, this synopsis. Insert the final ClinicalTrials.gov registration number and IRRID when available.
