# Supplementary material for: Immersive Virtual Reality Cognitive Stimulation for Delirium Prevention in Older Surgical Intensive Care Unit Patients With Cognitive Impairment: Protocol for a 3-Arm Randomized Pilot Trial
Source: JMIR Res Protoc. 2026 Nov 5;15:e105624. doi: 10.2196/105624 (PMC13740225; doi:10.2196/105624)
Supplement: Supplementary file 2 [file resprot-v15-e105624-s002.pdf]

Multimedia Appendix 1. SPIRIT 2013 Checklist

ReCognitionVR 2.0 randomized pilot trial protocol manuscript

Note. This checklist was completed for a JMIR Research Protocols submission describing a randomized pilot trial protocol. Items that require trial results are marked as not applicable for the protocol manuscript and should be completed in the future results manuscript.

SPIRIT item	Topic	Recommended item	Manuscript location	Protocol/manuscript text or summary	Status
1	Title	Descriptive title identifying the study as a randomized pilot trial protocol.	Title page	Immersive Virtual Reality Cognitive Stimulation for Delirium Prevention in Older Surgical ICU Patients With Cognitive Impairment: Protocol for a Three-Arm Randomized Pilot Trial.	Addressed
2a	Trial registration	Trial identifier and registry name.	Abstract; Trial Registration field; Declarations	ClinicalTrials.gov registration number NCT07641153	Addressed
2b	Protocol version	Protocol identifier and date.	Study Design and Methods; Ethics and Dissemination	PRO00041199, modified version 2 dated April 13, 2026.	Addressed
3	Funding	Sources and types of support.	Title page; Declarations	National Institute on Aging, 1K76AG088424-01A1.	Addressed
4	Roles and responsibilities	Sponsor, coordinating center, committees, and contributors.	Declarations; Acknowledgments; Methods	Author contributions, the coordinator role, the Safety Officer role, and study team roles are described.	Addressed
5a	Background and rationale	Scientific background and rationale for the trial.	Introduction	Delirium burden, nonpharmacologic prevention, cognitive stimulation, and VR rationale are described.	Addressed
5b	Choice of comparators	Rationale for control arms.	Methods: Intervention Arms; Discussion	Passive VR controls for immersion/headset exposure; nurse-led orientation reflects standard care.	Addressed
6a	Objectives	Specific objectives and hypotheses.	Abstract; Introduction; Outcomes	Feasibility as the primary objective; cognitive outcomes as secondary; delirium as exploratory.	Addressed
6b	Trial design	Trial design, allocation ratio, and framework.	Study Design and Methods	Single-center, three-arm, parallel-group randomized pilot trial with 1:1:1 allocation.	Addressed
7	Study setting	Study setting and sites.	Abstract; Study Design and Methods	Surgical intensive care units at Houston Methodist Hospital.	Addressed
8	Eligibility criteria	Inclusion and exclusion criteria.	Participants	Adults >=65 years with MoCA 15-24 after major surgery and expected ICU admission; key VR safety exclusions listed.	Addressed
9	Interventions	Interventions in sufficient detail for replication.	Intervention Arms; Development of ReCognitionVR 2.0; Figure legends	ReCognitionVR 2.0 cognitive stimulation, passive VR exposure, and nurse-led reality orientation are described.	Addressed
10	Outcomes	Primary, secondary, and exploratory outcomes.	Outcomes; Tables 2 and 3	Feasibility domains, cognitive outcomes, digital performance measures, and delirium incidence are defined.	Addressed
11	Participant timeline	Schedule of enrollment, interventions, and assessments.	Schedule of Assessments; Figure 1	Screening, postoperative days 1-3 intervention, postoperative days 1-7 delirium assessment, and discharge surveys are described.	Addressed
12	Sample size	Sample size rationale.	Sample Size	N=90, 30 per arm, selected for feasibility precision and planning estimates.	Addressed
13a	Allocation sequence generation	Method of random sequence generation.	Randomization and Blinding	Simple computer-based randomization using the REDCap randomization module.	Addressed
13b	Allocation concealment	Mechanism to conceal allocation.	Randomization and Blinding	Computer-generated allocation through REDCap after eligibility confirmation and baseline assessment.	Addressed
13c	Implementation	Who generates and implements allocation.	Randomization and Blinding; Methods	Implemented through REDCap with blinded outcome assessors; final operational details maintained in MOP.	Addressed
14	Blinding	Who will be blinded and how.	Randomization and Blinding	Participants cannot be blinded to VR exposure; outcome assessors remain blinded; participants blinded to the study hypothesis.	Addressed
15	Data collection methods	Plans for assessment and data collection.	Schedule of Assessments; Data Collection and Management	Standardized CRFs, REDCap entry, cognitive and delirium assessment training, and device-generated metrics.	Addressed
16	Data management	Data entry, coding, security, and quality.	Data Collection and Management	REDCap, unique identifiers, source data verification, query resolution, and protocol deviation tracking.	Addressed

17	Statistical methods	Analytic methods for primary and secondary outcomes.	Statistical Analysis	Descriptive feasibility estimates, mixed-effects models for cognitive outcomes, logistic regression for delirium if event counts permit.	Addressed
18	Data monitoring	Data monitoring committee or safety oversight.	Safety Monitoring	Independent Safety Officer and Data and Safety Monitoring Plan.	Addressed
19	Harms	Adverse event collection and reporting.	Safety Monitoring; Safety Medication and Intervention Modification	Expected VR-related symptoms, vital sign thresholds, session stopping rules, and SAE reporting.	Addressed
20	Auditing	Trial conduct auditing, if any.	Data Collection and Management	Source data verification, quality control review, REDCap query resolution, and protocol deviation tracking are planned.	Addressed
21a	Ethics approval	Research ethics approval.	Ethics and Dissemination; Declarations	Houston Methodist IRB approval status and protocol number are provided; final approval documentation to be uploaded if permitted.	Addressed
21b	Protocol amendments	Communication of protocol amendments.	Ethics and Dissemination; Appendix 3 synopsis	Modified protocol version and date are documented.	Addressed
22	Consent or assent	Consent and assent procedures.	Recruitment, Screening, and Consent; Capacity and Assent Procedures	LAR consent, capacity assessment, verbal assent, and behavioral dissent are described.	Addressed
23	Confidentiality	Confidentiality protections.	Data Collection and Management; Ethics and Dissemination	Unique identifiers, separate identifiable data, and institutional data safeguards.	Addressed
24	Conflicts of interest	Financial and competing interests.	Declarations	Competing interests and institutional IP status are to be completed by all authors.	Addressed
25	Access to data	Data access and data sharing.	Declarations; Data Availability	No participant-level data in protocol; future deidentified data sharing per institutional, IRB, and NIH policy.	Addressed
26	Ancillary and post-trial care	Care after trial or harm compensation.	Safety Monitoring; Intervention Modification	Clinical care retains priority; adverse events followed until resolution/stabilization.	Addressed
27	Dissemination policy	Plans for dissemination and authorship.	Ethics and Dissemination; Declarations	Peer-reviewed publication, scientific presentation, and ICMJE authorship.	Addressed
28-33	Informed consent materials and appendices	Additional protocol materials, appendices, and participant-facing materials.	Multimedia Appendices; Appendix 3	SPIRIT checklist, CONSORT-EHEALTH checklist, and approved protocol synopsis are provided.	Addressed

Prepared for submission with: Immersive Virtual Reality Cognitive Stimulation for Delirium Prevention in Older Surgical ICU Patients With Cognitive Impairment: Protocol for a Three-Arm Randomized Pilot Trial.
